# Epidemiological considerations on African swine fever in Europe 2014–2018

**DOI:** 10.1186/s40813-018-0109-2

**Published:** 2019-01-09

**Authors:** Erika Chenais, Klaus Depner, Vittorio Guberti, Klaas Dietze, Arvo Viltrop, Karl Ståhl

**Affiliations:** 1 National Veterinary Institute, Uppsala, Sweden; 2 Friedrich Loeffler Institute, Friedrich, Germany; 3 National Institute for Environmental Protection and Research, Rome, Italy; 4 Estonian University of Life Sciences, Tartu, Estonia

**Keywords:** ASF transmission, Wild boar, Pig, Contagiousity, Biosecurity

## Abstract

In 2007 African swine fever (ASF) arrived at a Black Sea harbour in Georgia and in 2014 the infection reached the European Union (EU), where it still expands its territory. ASF is a fatal viral disease affecting domestic pigs and wild boar of all ages with clinical presentations ranging from per-acute to chronic disease, including apparently asymptomatic courses. Until the detection of the first case inside the EU, infections in the current epidemic were mainly seen among pig farms with generally low biosecurity, and with incidental spill over to the wild boar population. In the EU, however, the infection survived locally in the wild boar population independently from outbreaks in domestic pigs, with a steady and low prevalence. Apart from the wild boar population and the habitat, the current epidemic recognizes humans as the main responsible for both long distance transmission and virus introduction in the domestic pig farms. This underlines the importance to include social science when planning ASF-prevention, −control, or -eradication measures.

Based on experiences, knowledge and data gained from the current epidemic this review highlights some recent developments in the epidemiological understanding of ASF, especially concerning the role of wild boar and their habitats in ASF epidemiology. In this regard, the qualities of three epidemiological traits: contagiousity, tenacity, and case fatality rate, and their impact on ASF persistence and transmission are especially discussed.

## Background

African swine fever (ASF) is a fatal viral disease of pigs, affecting domestic pigs and wild boar of all ages without sex predilections [1]. Depending on virus strain and immunological status of the animal, infection can lead to a wide range of clinical presentations varying from per-acute to chronic disease, including apparently asymptomatic courses [2, 3]. Infection with virulent strains typically causes per-acute to acute lethal ASF with signs including sudden death, high fever, hemorrhages in the skin and internal organs. The animals usually die within three to ten days after infection and the case fatality rate can reach 90% or more [4, 5].

In most cases, high titers of ASF virus (ASFV) can be found in the blood of infected animals from the time they develop clinical signs. Thus, transmission through contact with infected animals mainly happens once clinical disease is evident. Transmission can either occur directly through close contact with infectious animals or indirectly through ingestion of infected pork products or contact with fomites, and possibly via mechanical vectors [6]. In addition, the virus can be efficiently transmitted through the biological soft tick vector, genus *Ornithodoros* spp., where this is present. However, the *Ornithodoros* spp.is not considered to play a role in the epidemiology of ASF in the current epidemic in Central and Eastern Europe [7]. In absence of the tick vector, the most efficient way of virus transmission is via direct contact with blood from infected animals [8].

**Fig. 1:**
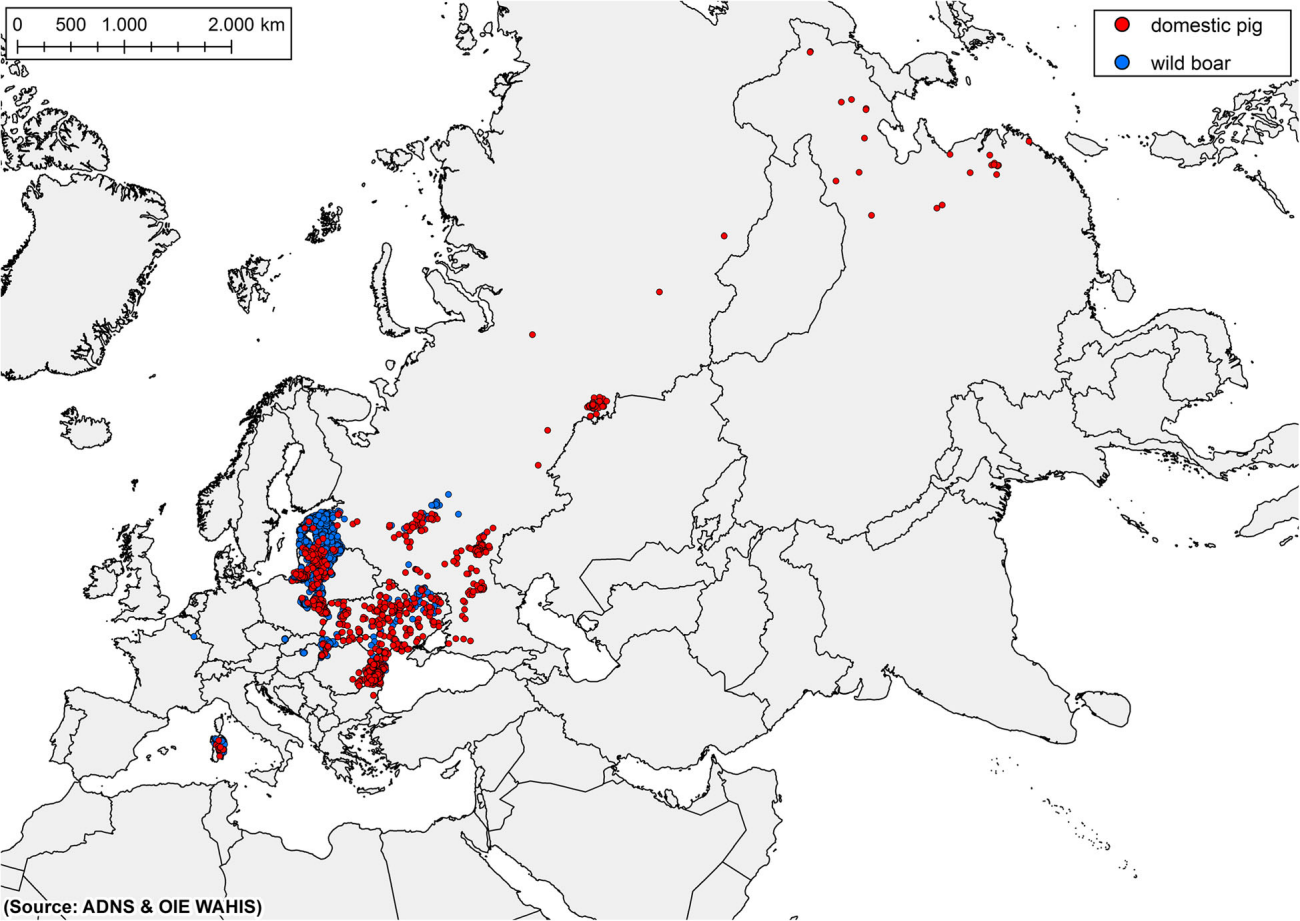
Notifications of cases in wild boar and outbreaks in domestic pigs in the European Union. Data extracted from the Animal Disease Notification System from January 2017 until September 2018

The ASFV strain in the current epidemic is a highly virulent strain belonging to the genotype II [9–13]. The epidemic started in Georgia in 2007 [14], and most probably originated from improper disposal of infected pork from a ship at the Black Sea harbour of Poti [15]. From Georgia the virus spread throughout the Caucasus and the Russian Federation (RF), where the disease subsequently became endemic [16, 17]. In July 2012, ASF was reported from Ukraine and in June 2013 it was notified by Belarus [5]. In January 2014 ASF reached the eastern borders of the European Union (EU) when the very first cases of infected wild boar were reported from Lithuania. In February the same year Poland reported its first cases, followed by Latvia in June and Estonia in September [18]. In the three Baltic States and Poland the disease has become endemic in the wild boar populations [19], whereas the sporadic outbreaks occurring in domestic pigs have been efficiently controlled preventing extensive secondary spread [20]. The latest countries affected in Europe are Belgium, Bulgaria, Czech Republic, Hungary, Moldova and Romania, all with cases in wild boar or outbreaks in domestic pigs in 2017 or 2018 (see Fig. 1). In the EU over 12.000 cases in wild boar and over 1.300 outbreaks in domestic pigs have been notified since 2014 (see Table 1). Regarding the wild boar cases it can be assumed that the real number is much higher since not all wild boar which succumb from the disease are found, tested and reported. Furthermore, reporting frequencies are also affected by different control measures and reporting incentives within the affected countries, e.g. by paying or not paying for hunting wild boar or reporting dead animals.

**Table 1:** ASF notification in domestic pigs and wild boar in the EU since January 2014 until September 2018

Year	Domestic pig holdings	Wild boar	Countries
2014	40	264	LV, LT, EE, PL
2015	42	1639	LV, LT, EE, PL
2016	48	2300	LV, LT, EE, PL
2017	124	3855	LV, LT, EE, PL, CZ, RO
2018	1123	4024	LV, LT, EE, PL, CZ, RO, HU, BG, BE
TOTAL	1377^a^	12,082	

^a^Out of these outbreaks 954 occurred in Romania

Data extracted from the Animal Disease Notification System. Italy/Sardinia is excluded and only cases and outbreaks caused by ASFV genotype II are summarized

This review aims to highlight some recent developments in the epidemiological understanding of ASF, especially concerning the role of wild boar and their habitats in ASF epidemiology. The review is based on experiences, knowledge and data gained from the current epidemic in the parts of Central and Eastern Europe where wild boar are implicated.

## Epidemiology

**Fig. 2:**
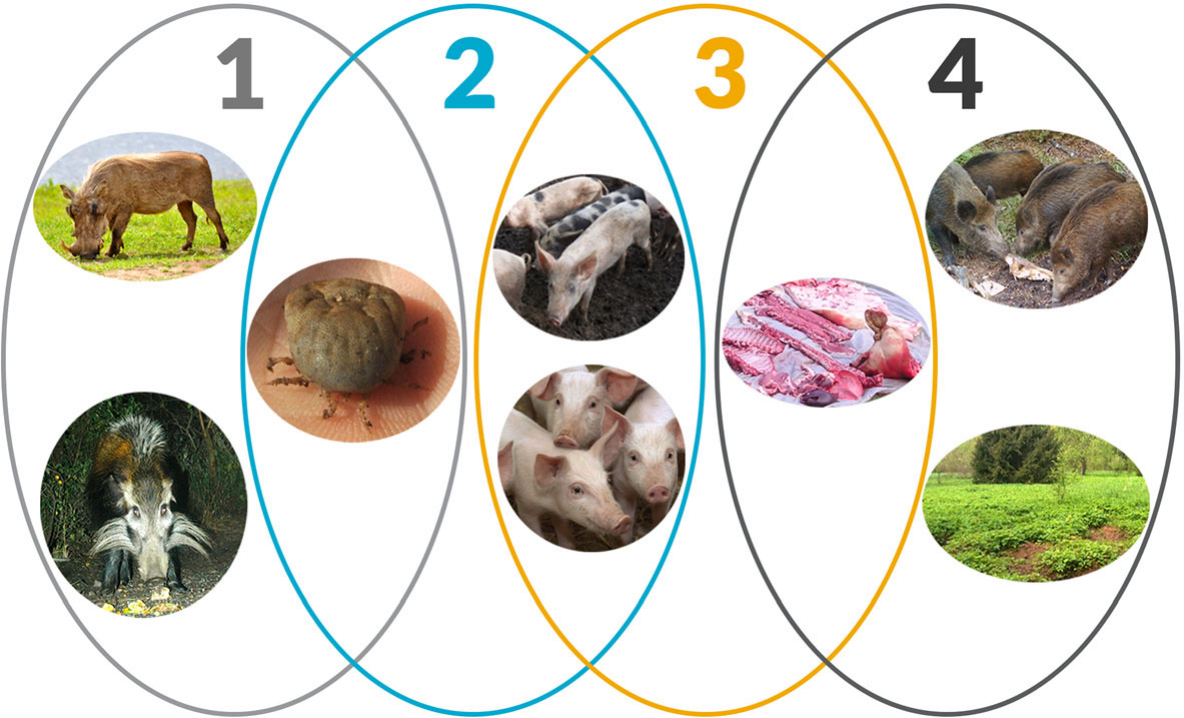
The four transmission cycles of ASF with the main transmission agents depicted. The role of the bushpig in the sylvatic cycle remains unclear. Illustration: Magdalena Hellström, photographs by Erika Chenais, Klaus Depner and Karl Ståhl. The figure was originally published in Emerg Infect Dis 24, 810

Until recently ASF epidemiology was described as comprising three independent epidemiologic cycles (sylvatic, tick–pig, and domestic), involving soft *Ornithodoros* spp. ticks, wild African pigs (mainly warthogs), domestic pigs, and pig-derived products such as pork [21]. In the sylvatic cycle, ASFV circulates between the natural reservoirs of the virus (i.e., warthogs and soft ticks), without causing disease in the vertebrate host [22]. This ancient cycle is the origin of the tick–pig cycle and the domestic cycle, and thus the origin of ASF as a disease. In the tick–pig cycle, the virus is mostly transmitted among domestic pigs, with the ticks serving as a reservoir allowing the virus to persist locally in the environment [23]. This cycle has been described in parts of sub-Saharan Africa, but also played an important role for the persistence of the disease during the epidemic on the Iberian Peninsula in the ‘60s and ‘70s of the past century [24]. In the domestic cycle, which is the cycle involved in the vast majority of outbreaks of ASF globally [6], the virus is transmitted among domestic pigs, or from pig products to domestic pigs. This cycle does not involve the natural reservoirs. The epidemiological pattern observed from the current ASF epidemic in Central and Eastern Europe, however, does not match any of the previously described cycles. Rather it revealed an additional epidemiological cycle (see Fig. 2) including Eurasian wild boar (*Sus scrofa*), the wild boar habitat and their carcasses. This fourth cycle has been named the wild boar–habitat cycle [25]. This cycle is characterized by both direct transmission between wild boar, and indirect transmission via the habitat. The habitat contamination through ASFV infected wild boar carcasses offers possibilities for new infections depending on landscape, time, season and carcass decomposition [26]. Environmental persistence of the virus is favored by cold and moist climate.

## Transmission

From the start of the current epidemic in 2007, until the detection of the first case inside the EU in 2014, infections were mainly seen among pig farms with generally low biosecurity, and with incidental spill over to the wild boar population. At that point in time it was predicted that the disease would spontaneously fade out from the local wild boar population as soon as the disease was under control in the domestic pig population, due to the high case fatality rate and the absence of long-time carriers [27]. However, in the ecological context which prevailed in Poland and the Baltic states this epidemiological hypothesis proved to be wrong. The infection survived locally in the wild boar population independently from outbreaks in domestic pigs, with a steady and low prevalence below 5% and a local transmission speed of 2–5 km/month [28]. In addition to the local transmission within the wild boar population, long distance jumps responsible for disease incursion into areas far from known infected regions occurred. In the EU the most recent events of such long distance ASF spread took place in the Czech Republic (Zlin area), Poland (area of Warsaw), Hungary and Belgium. These recently infected areas were each several hundreds of kilometers away from previously known infected regions. Likewise, in early March 2017 an ASF outbreak was reported from the Irkutsk Region in the RF, close to the Mongolian border, more than 4000 km away from the nearest outbreaks in the European parts of the RF [29], and more recently in August 2018 a first outbreak was reported from the province of Liaoning in north-eastern China [30]. The described long-distance jumps are most probably attributable to human activities (anthropogenic factors), e.g. transport of contaminated meat or meat products ending up as waste or kitchen leftovers either in pig stables or in natural environments inhabited by wild boar [31–33]. These examples demonstrate that due to the anthropogenic factors, ASF has a huge capacity of transboundary and transcontinental spread [34].

Human activities have been identified as main drivers of disease transmission in the domestic epidemiological cycle of ASF in other contexts [35]. Likewise, social and economic factors such as poverty level, herd size and gross income from the pig production have been associated with ASF outbreaks in the domestic cycle [36]. The same authors also showed that smallholder farmers in some contexts seem to have high levels of knowledge regarding ASF, but that other factors than knowledge will guide decisions affecting disease transmission, such as trade and slaughter. Livelihood circumstances often prevent farmers from executing preventive actions they are aware of, and may even force farmers to take actions that promote disease transmission. In addition, the decisionmaking process affecting disease dynamics and the possibility to control disease spread is much more complex than what scientists have previously acknowledged, including not only economic aspects, but factors such as cultural identity, tradition, peer pressure, quality of the relationship with authorities and animal welfare aspects [37, 38]. In the current epidemic, social as well as infrastructure networks have been proved to affect trade patterns [39, 40], and thus most probably, also disease transmission. Further, control measures imposed by governments, such as stamping out without sufficient compensation and trade restrictions, might be counteractive in many contexts [41]. Trade will not necessarily cease after a quarantine has been put in place, but move to uncontrolled markets, or more distant points of sales, conceivably increasing disease transmission [42]. Such trade and quarantine factors might have been the drivers behind several transboundary outbreaks in the Caucasus region, where pork prices dropped as outbreaks occurred, increasing cross-border trade [43].

## Contagiousity

In textbooks ASF is often described as a highly contagious disease with high mortality [44, 45]. The case fatality rate (i.e. the proportion of infected individuals that die from a disease within a certain time period) related to highly virulent ASFV in affected populations of domestic pigs and wild boar is indeed high, often reaching 90–100% [46]. When pigs show clinical sign of ASF they have high viral loads in all body secretions, with particularly high levels in blood [47, 48]. If pigs are not euthanized before this stage, and especially if they are in an environment allowing for close and frequent contacts with other pigs, blood exposure and possibly cannibalism, the potential for environmental contamination is high [49]. In these specific situations, ASF shows patterns of a highly contagious disease. On the other hand, in a context where cases are detected early and disease control measures (including depopulation) implemented swiftly, the contagiousity will be low, as supported by the analyses of the domestic pig outbreaks in the current epidemic [20]. This is probably to some degree a mirror of effective disease surveillance and control. As an example, on several occasions during the current epidemic only one or few diseased or dead animals were present on affected farms at the time of suspicion [50, 51]. Likewise, on these occasions, animals which were in direct contact with ASF-positive pigs tested negative although they had been in the same stable for more than one week [50, 51]. This observation indicates that under field conditions, ASFV transmission between animals can be a slow process.

Low contagiousity of ASF has also been demonstrated under experimental conditions. Pietchmann et al. (2015) conducted a study to assess the risk of chronic disease and the establishment of carriers upon low-dose infection of pigs and wild boar with ASFV genotype II. Although the study was not primarily aiming at establishing the contagiousity of ASF, meaningful findings regarding this matter were obtained. The experimental animals were inoculated oro-nasally with low doses of ASFV (10–100 haemadsorbing units) and only the weakest animals (2 out of 12 wild boar) became infected. However, during the course of the experiment the initially non-infected animals picked up the infection by direct contact with the infected ones, after these had succumbed to clinical disease. The authors concluded that very low doses of virus are linked to moderate contagiousity leading to a scattered onset of clinical signs [8].

It is conceivable that similar scenarios take place in field situations in wild boar populations or in domestic pig herds. Due to the low contagiousity, which might be due to a low dose exposure and/or oral transmission route, the initial mortality within an epidemiological unit is rather low regardless of the high case fatality rate. From Latvia, Lamberga et al. (2018) reported how in a large commercial farm with 5000 pigs affected by ASF, the spread of the virus within the farm was slow [52]. Within the first weeks of infection, the ASF related mortality did not exceed the usual farm mortality and it took more than one month until ASF was suspected. Similar observations of slow virus spread within affected pig farms have been made in Estonia [51, 53].

## Virus survival in the environment

ASFV has been shown to persist in meat from infected pigs when stored for several months at around 4 °C; in skin fat for 300 days; in salted, dried meat for up to 120 days; and in ham in brine for up to 180 days [22, 54–57]. At 4 °C the virus persists for over a year in blood, several months in boned meat and several years in frozen carcasses [58, 59]. Given all this tenacity data, it is easy to understand why and how virus contaminated meat and meat products have played a crucial role in the transmission and epidemiology throughout the history of ASF. It explains how the virus can travel from one country to the other or from one continent to the next. Particularly pigs in backyard systems where swill feeding is still common practice, are under major risk to become infected via this route. All virus escapes from Africa to other continents have been linked with transport of contaminated pork with airplanes or ships e.g. the first outbreaks in Portugal and Spain in 1957 and 1960 [60] and the more recent outbreak in Georgia in 2007 [14]. Furthermore, the virus also survives the process of putrefaction [4, 6]. It has been shown that ASFV genomic material can be detected with PCR as long as tissue samples can be obtained from carcasses left in the field [61]. However, as PCR and not virus detection was used, no conclusions on the survival of the virus or its infectivity can be drawn from that study. The ability to remain infective after putrefaction is of particular importance for wild boar carcasses that remain in the environment until total decomposition. Probst et al. (2017) monitored the behavior of free ranging wild boar towards carcasses of their own conspecifics. The direct contact with wild boar carcasses consisted mostly in sniffing and poking on the carcass, scavenging was not observed. It was concluded that all these types of contact still represent a risk for infection [26]. In this regard, the low contagiousity of ASFV is contrasted by the high tenacity. Contaminated wild boar carcasses might facilitate virus persistence for months or even years within a region, significantly influencing the course of an ASF epidemic. Even if the probability of infection for each contact is low, the long infectious period will allow the virus circulation to be maintained. Further complicating this process, in the study by Probst et al. (2017) wild boars were more interested in the soil underneath and surrounding the carcass than in the wild boar carcasses themselves. During the process of carcass decomposition, potentially ASFV-containing carcass material penetrates the soil underneath and in the vicinity of the carcass. Soil samples taken from places, where ASF positive carcasses had been found, were PCR-positive several days or weeks after the carcass had been removed, although, no viable virus could be isolated (unpublished data, Arvo Viltrop). Therefore, soil from underneath the carcasses contaminated with ASFV may also play a role in the epidemiology of ASF. In experimental studies it has been shown that ASFV remains infectious in forest soil up to 112 days [57]. The same authors also showed experimentally that the virus may remain infectious in water from a lake up to 50 days during summer and 176 days during winter and that the virus survived over two months on wooden boards and three months on bricks buried into the earth. It should be noted that these experiments were based on parenteral inoculation of the test materials which requires much lower virus dose for infection than the oral route, which is more likely to occur in nature. As for direct contact, the probability that wild boars acquire the infection via soil will mainly depend on the susceptibility of the animals and the type, frequency, and intensity of contacts. Due to the short phase of clinical signs and associated virus excretion; wild boar behavior, ecology and population density; and the tenacity of ASFV in carcasses, the spread of ASFV through carcasses is considered to be more important than direct contact with live infectious animals for wild boar [18, 62].

## Biosecurity, the most effective tool for controlling ASF

### Farm biosecurity

Good farm biosecurity is considered to be the most important tool for preventing ASF introduction on a holding [63]. Many ASF field studies report on biosecurity shortcomings, and mention this inadequacy as a critical factor for virus introduction via links to infected wild boar and swill feeding [50, 64]. Roughly, farm biosecurity can be split into two components; (i) biosecurity hardware, envisaging the quality of buildings, fences, equipment, roads, gates, etc. and (ii) biosecurity software, which can be seen as a mindset, dealing with the managerial procedures related to human activities, hygiene regime, education of personnel, participatory development of biosecurity to suit the local conditions on site etc. For example, a pig farm with excellent biosecurity hardware (proper buildings, fences, hygiene barriers, personal equipment for visitors) can still become ASF-infected if people do not follow the stipulated procedures, and vice versa. The backyard pig sector represents a huge challenge in this regard, in particular due to its heterogeneity, and special efforts should be made to improve implementation of biosecurity and raising awareness to promote early detection of ASF. Nevertheless, in Estonia the larger herds had significantly higher risk of experiencing an ASF outbreak compared to smallholders [65]. This could be the result of somewhat specific situation in Estonia, where swill feeding and animal movements, otherwise tending to be the main risk factors for back yard holdings, did not play any significant role in spread of the infection. In a situation where environmental contamination is suspected to be the major cause of disease introduction via contaminated vehicles and people, the frequency and intensity of contacts between surrounding environment and the farm premises are likely more important, and large farms become more vulnerable.

**Fig. 3:**
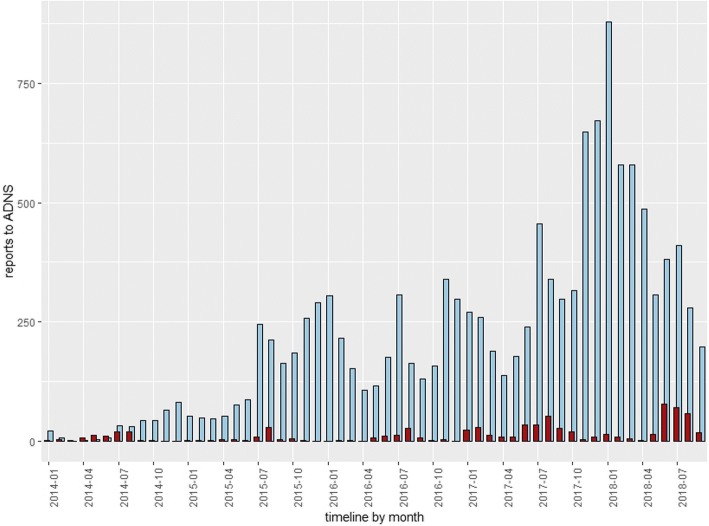
Number of notifications of cases in wild boar and outbreaks in domestic pigs in the European Union from 1st January 2014 until 25th September 2018. Data extracted from the Animal Disease Notification System. Blue bars are cases in wild boar and red bars are outbreaks in domestic pigs

**Fig. 4:**
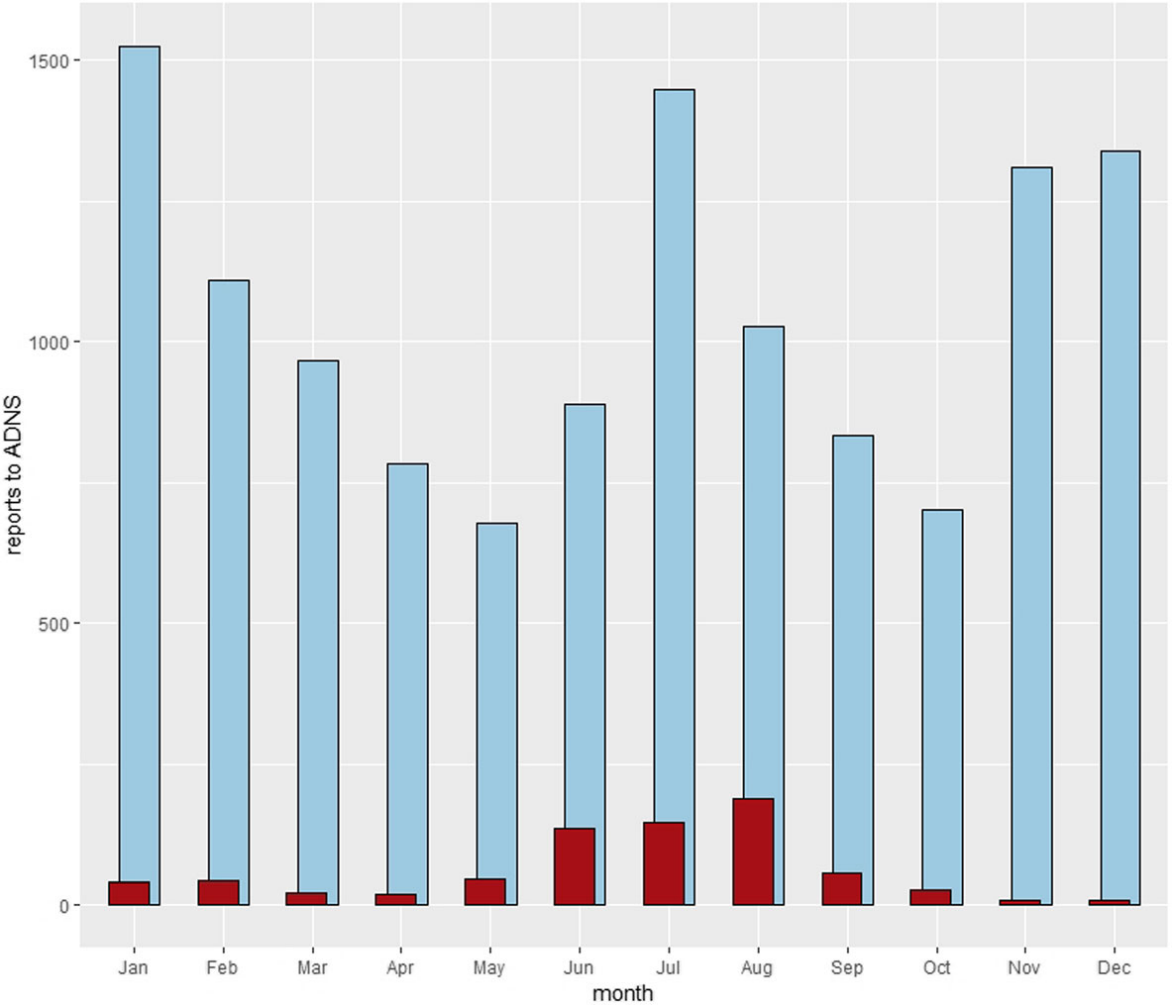
Number of notifications of cases in wild boar and outbreaks in domestic pigs in the European Union from 1st January 2014 until 25th September 2018, aggregated per month. Data extracted from the Animal Disease Notification System. Blue bars are cases in wild boar and red bars are outbreaks in domestic pigs

Incursion of ASF into the domestic pig population is often of anthropogenic nature, happening as spillover from affected wild boar populations in the immediate farm neighborhood, or through the unintentional introduction of contaminated material onto the farm premises [65]. Having said that, seasonal peaks of cases in wild boar occur bi-annually around June–July and November–December, and for outbreaks in domestic pigs annually from June to August (see Figs. 3 and 4). Seasonal variations in wild boar demography and ecology, farming and recreational activities in farm land and forests, temperatures and other climate factors, as well as in the activity and abundance of potential mechanical vectors have been brought forward as explanations for this pattern, which until now remains unexplained [65–67].

The vast majority of outbreaks that have been reported from the Baltic states and Poland have been classified as primary outbreaks with only very few secondary outbreaks [20]. Due to the absence of vaccines and drugs for prophylactic measures and treatment, implementing biosecurity measures at farm level remain the key component of ASF prevention and control [63]. As pig production systems are heterogeneous not only in size and degree of specialization, but also regarding the level of professionalization of the farm staff, realistic options for implementing biosecurity measures are diverse [68, 69]. However, observational data [70] as well as modelling approaches [71, 72] show that, despite this diversity, the implementation of basic biosecurity measures has substantial influence in reducing the persistence and spread of ASF in domestic pig production systems. Despite this, ASF continuous to occur in areas where the disease is known to all stakeholders. To understand this paradox, we need to look at biosecurity not only from the angle of hardware biosecurity, meaning infrastructure and identified procedures, but also from the software biosecurity perspective. Acknowledging a mindset or philosophy component as part of the definition of biosecurity allows enforcing authorities to better comprehend the importance of promoting measures that match farming realities of a diversified group of production systems. Putting regulations in place that request farms to implement biosecurity according to their level of production (e.g. in the European Union as part of a regional ASF strategy [73]), has been a useful step working on the hardware component of biosecurity. However, in particular in backyard production systems where risky production practices such as swill feeding remain, the software component appears to require additional efforts to become a sustainable component of ASF prevention.

### Biosecurity during hunting and carcass removal

During the last 40 years the geographical range of wild boar has expanded and population densities increased [74]. This has happened despite hunting. Hunting management might even have contributed to these trends through sustaining winter-feeding, avoidance of shooting adult females, and hunting bags well below the natural recruitment rate of the species [75]. Currently thousands of infected wild boar are found dead or are hunted each year in the more than 200.000 km^2^ that are under restriction due to ASF within the EU. Recent experiences show that carcass detection is the most important tool to detect geographical spread in wild boar, and that carcass removal (including sampling and safe destruction) seem essential to reduce transmission in infected areas [67]. Due to the characteristics of the virus, there is a risk of local ASF-persistence through carcasses and offal, as well as for indirect transmission through contaminated tools and cars used during hunting [21]. Therefore, a model for management of infected areas including core and buffer areas with no hunting and continuous carcass removal surrounded by an area with intensive, restricted hunting, has now been proposed [75]. In this model, hunting in the surrounding areas is permitted only for hunters trained on sampling and biosecurity measures. Some examples of measures to improve biosecurity during hunting are using leak proof vessels (e.g plastic troughs) to carry/drag carcasses out of the woods; limiting the use of private cars in infected areas, including a ban on transporting hunted animals in private cars; organization of a dressing area that limits the blood contamination of hunting tools and vehicles; washing and disinfecting all tools used to dress wild boar after use and leaving these in the infected area; and storing offal in biosafe containers [75]. Individual identification of hunted wild boar before storing and testing, keeping hunted wild boar in the area until tested negative for ASF, veterinary supervised disposal of all stored carcasses and cleaning and disinfection of the dressing area in case of a positive test outcome are other measures necessary for biosafe hunting in infected areas [75].

## Conclusions

The qualities of the three epidemiological traits contagiousity, tenacity, and case fatality rate make ASFV efficient in both persistence and transmission. The high tenacity ensures long term persistence in the environment, high case fatality rate makes the virus largely available, and the relatively low contagiousity prevents the complete depletion of the host population. The interaction of these three parameters maximize both local persistence and geographical spread of the virus making its eradication a challenge. The disease does not show a typical epidemic pattern with either self-limiting localized epidemics or wider spread through an epidemic wave [76]. Both these patterns would probably require higher contagiousity. The patterns usually observed in endemic settings, with a constant circulation or presence of pathogens in the target population [76], is also not observed. With a high case fatality rate and the probable absence of a long-lasting carrier status, ASFV cannot be maintained independently in an active circulation over a longer time despite the high reproductive capacity of wild boar. This leaves us the epidemiological scenario of a reservoir-facilitated perpetuation leading to an endemic state. With the absence of the reservoir hosts, African wild suids or O*rnithodoros* spp. ticks, the habitat as such, including the contaminated carcasses have to be considered as pathogen reservoir leading to the observed endemic setting with extended transmission intervals. Apart from the wild boar population and the habitat, the current epidemic recognizes humans as the main responsible for both long distance transmission and virus introduction in the domestic pig farms. Thus it becomes crucial to include social science when planning prevention-, control-, or eradication measures. By considering only the biological particularities of the disease, contagiousity, tenacity and case fatality rate, but ignoring the human aspects, the epidemic will not be controlled.
